# Labor Analgesia in Brugada Syndrome and the Importance of Contingency Planning

**DOI:** 10.1155/2022/8940704

**Published:** 2022-07-15

**Authors:** Shayla Mena, Ana Costa, Michelle DeLemos, Joy Schabel, Morgane Factor

**Affiliations:** Department of Anesthesiology, Stony Brook University Renaissance School of Medicine, 101 Nicolls Road, Health Sciences Center, Level 4, Stony Brook, NY 11794-8434, USA

## Abstract

Brugada syndrome is an autosomal dominant disorder that affects cardiac sodium channels and predisposes patients to an increased risk of sudden cardiac death. Obstetric anesthesia management in patients with Brugada syndrome poses a challenge due to the prevalence of local anesthetic use for labor analgesia or cesarean section. However, central neuraxial techniques and local anesthetics have been used safely in parturients with this syndrome and may be offered to patients during preadmission counseling. We present the case of a primigravida who opted for further labor analgesia via a combined spinal-epidural technique. To our knowledge, this is the first case report of a lidocaine infusion administered via an epidural catheter to a laboring parturient with Brugada syndrome. We further discuss the use of local anesthetics, other medications, and central neuraxial techniques in those with Brugada syndrome to assist anesthesiologists caring for expectant mothers.

## 1. Introduction

Brugada syndrome is caused by a mutation in the *SCN5A* gene, has variable penetrance, and is diagnosed based on distinct electrocardiogram (ECG) changes either at rest or with sodium-channel blockade [1]. A parturient diagnosed with Brugada syndrome presented to our preoperative services clinic in the third trimester. She desired an attempt at a natural delivery but requested further management if her labor pain became intolerable. The literature was reviewed with the patient, and an anesthetic plan was made for management of labor pain or an unexpected cesarean section. The patient was ultimately admitted for induction of labor after 40 weeks' gestation. Eight hours after induction and with labor pain unalleviated by intravenous (IV) butorphanol, the patient elected for further analgesia. She received a combined spinal-epidural (CSE) with intrathecal bupivacaine and fentanyl, and an infusion of lidocaine, fentanyl, and epinephrine via an epidural catheter. The patient delivered shortly after initiation of the epidural infusion, and a serum lidocaine concentration was measured an hour after it was discontinued. The patient remained asymptomatic throughout her hospital stay and was safely discharged on postpartum day two. Preoperative communication with the patient's cardiologist, obstetrician, and an obstetric anesthesiologist allowed for a multidisciplinary approach based on the available medical literature.

## 2. Case Presentation

**Figure 1:**
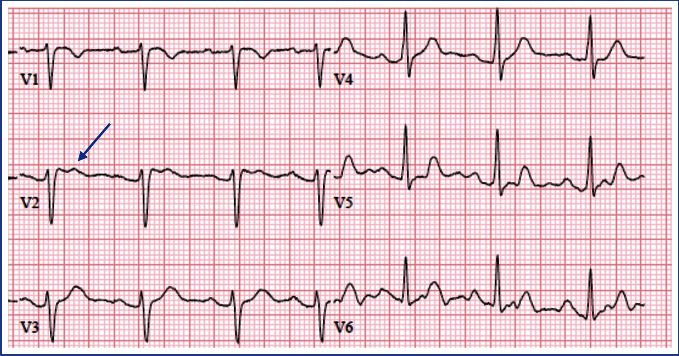
Patient's ECG demonstrating type II Brugada ST-segment elevation and a “saddleback” appearance in V2.

A 31-year-old G1P0 parturient with a past medical history of Brugada syndrome (diagnosed with procainamide testing and identification of the *SCN5A* mutation), Ehler-Danlos syndrome, and diet-controlled gestational diabetes mellitus presented to our preoperative services clinic upon recommendation by her obstetrician. Her family history was significant for Brugada syndrome without sudden cardiac death (SCD). Her aunt and uncle remained asymptomatic, while her father had an implantable cardioverter defibrillator (ICD) placed for syncopal episodes. The patient did not have an ICD and was also asymptomatic. Of note, her aunt had multiple, uncomplicated pregnancies and the patient had upper-extremity orthopedic surgeries without complications. She had also consulted with a cardiologist who specialized in parturients with cardiac comorbidities prior to conception. An echocardiogram, Holter monitor test, and electrophysiology study for inducible arrhythmias were all normal. Her baseline ECGs demonstrated normal sinus rhythm, with variable ST elevations of 0.5 mm in leads V1 and V2 and the “saddleback” T wave morphology of Brugada type II (Figure 1).

Communication between the patient's obstetrician and cardiologist determined that she was expected to deliver at term or be scheduled for induction of labor after 40 weeks' gestation. Following consultation with the patient's cardiologist and an obstetric anesthesiologist, a detailed anesthetic plan was formulated and discussed with the patient. This plan was subsequently distributed to all labor and delivery anesthesiologists and obstetricians at our institution. The patient expressed a desire to attempt a natural vaginal delivery, and the use of medications would be minimized. If the patient requested further analgesia during labor, she would receive IV butorphanol with escalation to patient-controlled analgesia (PCA) with remifentanil. If her pain was not adequately treated, she agreed to a CSE with the understanding that an epidural infusion would only be utilized if she had not delivered during the portion of the spinal anesthetic. This would minimize the amount of local anesthetic used and the risk of a high spinal. Due to construction work on the labor and delivery unit's scavenging and ventilation system, nitrous oxide (Nitronox™) could not be offered to the patient during her admission.

At 40 weeks and two days of gestation, the patient was admitted for induction of labor. She received a vaginal insert of 10 mg dinoprostone and was placed on continuous telemetry monitoring in addition to standard blood pressure, maternal heart rate, and fetal heart rate monitoring. Over the following eight hours, she received three IV doses of 2 mg butorphanol, which were ineffective at providing analgesia. The patient ultimately reported 10/10 pain and requested further management. She was examined at the time and found to be ruptured, one cm dilated, 75% effaced, and at -four station. At that time, the patient denied further escalation to a PCA and opted for a labor epidural. After reiteration of the benefits and risks of central neuraxial techniques, an automatic defibrillator and crash cart were brought into the room. A CSE was subsequently performed at the L3-L4 level with a Braun Periflex® 17 gauge Tuohy needle and 25 gauge Pencan spinal needle. The procedure was successful on the first attempt with loss of resistance to air at 6 cm. There were no paresthesias or bleeding noted during any portion of the procedure. For the spinal anesthetic, a mixture of 20 *μ*g fentanyl and 1.25 mg 0.25% bupivacaine was administered. A Braun Periflex® 19 gauge epidural catheter was then threaded and secured at 10 cm with dry occlusive dressing and tape. After monitoring vital signs for 15 minutes, the patient reported pain relief to 3/10 and was hemodynamically stable. An obstetric nurse remained at the bedside for one hour after the procedure in the event the patient developed distressing symptoms.

One hour later, however, the patient complained of reoccurring 10/10 pain and pelvic pressure. An examination revealed 10 cm cervical dilation, 100% effacement, and zero station. The epidural catheter was then aspirated and tested with three mL 1.5% lidocaine with 1 : 200,000 epinephrine, diluted in normal saline to a total of eight mL. After a negative test dose, an additional two mL of 1.5% lidocaine with epinephrine 1 : 200,000 diluted in normal saline to seven mL was administered. A specially formulated epidural infusion of 0.25% lidocaine, 1 : 200,000 epinephrine, and 3 *μ*g/mL fentanyl was then initiated. It was set at a rate of seven mL/hr, with a demand dose of five mL every 10 minutes to an hourly maximum of 27 mL. Since two test doses were diluted in normal saline, a total of 15 mL was administered and considered an appropriate loading dose. The patient reported improvement in her pain bilaterally and delivered a healthy infant one hour and 58 minutes following initiation of epidural analgesia. The infusion was stopped immediately after delivery, and the total amount delivered (34 mL) was recorded. A serum lidocaine level of 0.8 *μ*g/mL (toxic level >5 *μ*g/mL) [2] was obtained one hour following discontinuation. The patient was admitted to postpartum and remained near close access to an automatic defibrillator and crash cart. She remained on telemetry for 24 hours and reported no symptoms throughout the postpartum course. She was discharged home on postpartum day two.

## 3. Discussion

**Figure 2:**
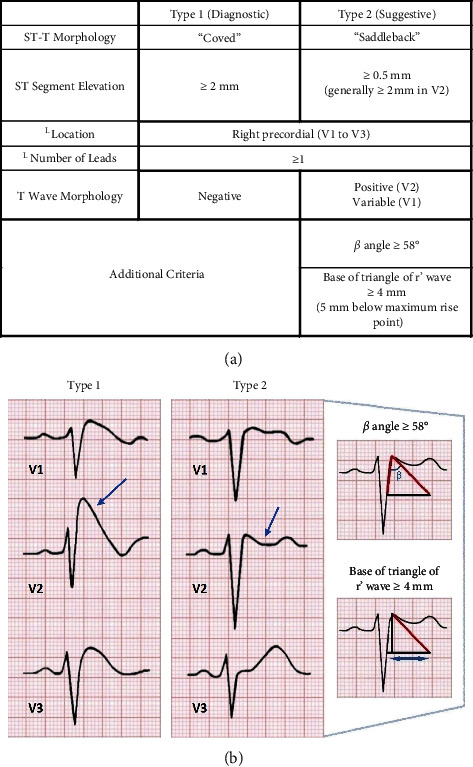
(a) Distinguishing characteristics of the two types of Brugada syndrome patterns. (b) Example ECG tracings of the two types of Brugada syndrome patterns, demonstrating the “coved” and “saddleback” ST-T morphology of type I and type II, respectively.

Brugada syndrome is an autosomal dominant genetic disorder of variable expression, commonly affecting the *SCN5A* gene, which encodes the *α* subunit of the cardiac sodium channel [3]. It leads to an increased risk for ventricular tachyarrhythmias and sudden cardiac death (SCD), with clinical manifestations ranging from chest discomfort, nocturnal agonal respiration, and syncope [4, 5]. The prevalence is highest in Asians, followed by Caucasians and Hispanics with a worldwide prevalence of 6.1 per 1000 [5]. Echocardiograms in patients with Brugada syndrome typically show a structurally normal heart, but ECG patterns may vary (Figure 2). The diagnostic Brugada ECG pattern, type I, shows “coved” ST-segment elevation in two or more anteroseptal precordial leads (V1–V3) [1]. The type II Brugada pattern is characterized by ≥0.5 mm ST-segment elevation in ≥ 1 right precordial lead (V1–V3), followed by a convex, “saddleback” ST and positive T wave morphology in V2 [1]. Of note, the ECG pattern of Brugada syndrome may be dynamic, presenting with normal electrical activity at times and unmasked by the administration of sodium channel blockers such as ajmaline, flecainide, and procainamide [6]. These sodium channel blockers can be used to diagnose Brugada syndrome in those with normal ECG patterns.

The most alarming manifestation of Brugada syndrome is a lethal ventricular tachyarrhythmia leading to SCD. Placement of an ICD is the definitive treatment of Brugada syndrome but is recommended only in patients at the highest risk, such as those who have survived cardiac arrest, have a history of syncope likely caused by ventricular arrythmias, or develop ventricular arrythmias with electrophysiologic provocation [7]. ICD placement is not indicated for asymptomatic individuals or on the basis of a family history of SCD [8]. Indeed, event rates in asymptomatic Brugada patients are low and a family history of SCD is not a predictor of future cardiac events [9]. When comparing female patients against male patients, women have a lower event rate (0.7% vs. 1.9% per year) and the only identified risk factors for future arrhythmic events are previous sinus node dysfunction and presentation as SCD [10].

Given that our patient remained asymptomatic since her diagnosis, her overall risk of a cardiac arrythmia during admission was low. During preadmission counseling, this was discussed with the patient as well as the precautions that would be utilized (e.g., communication to labor and delivery staff regarding safe medications to use, access to a crash cart and automatic defibrillator). While the patient expressed the desire at a natural delivery, further options for labor analgesia were reviewed. The patient was offered systemic opioid therapy followed by a labor epidural. The use of other nerve blocks such as a paracervical or pudendal nerve block would have carried a higher risk of fetal and maternal absorption of local anesthetic and thus not recommended [11]. A remifentanil PCA has been used for labor analgesia in Brugada syndrome [12] and was recommended over a fentanyl PCA owing to its rapid metabolism and decreased fetal effects [13, 14]. Finally, due to the higher risk of maternal morbidity and mortality with general anesthesia for cesarean section, the patient was counseled that in the event of an urgent or emergent cesarean delivery regional anesthetic techniques would be attempted before general anesthesia [15, 16].

There have been multiple reports on the safety of general anesthesia in patients with Brugada syndrome, with fewer reports on the use of regional anesthesia in parturients [17]. Although local anesthetics exert their action via sodium channel blockade, they have been used for regional anesthesia in expectant mothers. The current literature includes cases where local anesthetics and central neuraxial techniques were used safely in parturients with Brugada syndrome (Table 1). Theoretically, since the intrathecal dose needed to provide analgesia is minimal and there is limited systemic absorption, local anesthetics can be administered as a spinal anesthetic. However, their use should be restricted to the minimum necessary doses, including for epidural infusions. Indeed, while bupivacaine has been used intrathecally [18, 19], it has been implicated in the induction of a Brugada-type ECG pattern when administered via epidural catheter [20]. Ropivacaine has been used as an epidural infusion in patients with Brugada syndrome [21, 22], although one patient developed ventricular tachycardia after bilateral paravertebral blockade with Ropivacaine [23].

Considering the availability of other local anesthetic agents for epidural analgesia, and that labor analgesia often entails a prolonged infusion of local anesthetic, we opted to avoid our typical epidural infusion of bupivacaine and fentanyl as well as a ropivacaine infusion in this patient. Evidence suggests that lidocaine may be a safer agent for regional anesthesia in Brugada patients due to an inability to provoke ST-segment elevation following a bolus dose [29]. One must also consider its use as an antiarrhythmic agent and that epinephrine decreases lidocaine's systemic absorption while increasing the threshold for toxicity. Furthermore, intrathecal epinephrine produces analgesia even in the absence of local anesthetic, likely related to a spinal *α*-adrenergic mechanism [30].

During the preoperative assessment, we discussed performing a CSE and planned on using 0.25% bupivacaine and fentanyl, followed by an epidural infusion of 0.25% lidocaine, epinephrine, and fentanyl. Our institution's pharmacy was alerted to the patient's case to ensure preparation of a preservative-free infusion. The purpose of using intrathecal bupivacaine was to maximize the total duration of spinal analgesia. An epidural infusion would only be utilized if the patient's spinal anesthetic wore off and she had not delivered. During the infusion, serum lidocaine levels were to be measured every four hours, with discontinuation occurring if the patient developed symptoms of local anesthetic toxicity (e.g., circumoral numbness, tinnitus, dizziness, etc.) or cardiovascular complications (e.g., chest pain, palpitations, shortness of breath, etc.). She would remain under close observation for the first hour.

If the patient required a cesarean section and had a working epidural, 20 mL of 3% chloroprocaine or 2% lidocaine with 1 : 200,000 epinephrine would have been used to attain a surgical level. If the patient did not have a working epidural and required an urgent or emergent cesarean, a spinal anesthetic with hyperbaric bupivacaine (to a maximum of 12 mg), fentanyl (10–15 *μ*g), and morphine (150 *μ*g) would have been administered. If regional anesthesia was not feasible or if the spinal anesthetic failed, etomidate or propofol could be used sparingly [31], while ketamine would be avoided as an induction agent for general anesthesia [32]. Currently, the literature suggests that induction doses and maintenance dosing of propofol for short periods of time may be considered safe, while a prolonged infusion in a critically ill patient may unmask Brugada electrocardiographic features [33].

If the patient required induction of general anesthesia, neuromuscular blockers and their reversal agents would have been safe to use during the anesthetic [34, 35]. However, sugammadex may have presented a superior choice as a reversal agent, since neostigmine can cause ST-segment elevation in a dose-dependent manner [36]. Ergonovine is considered arrhythmogenic in Brugada syndrome, [37] and thus would have been used after other uterotonic agents were exhausted. Various medications and their safety profile were reviewed amongst the nursing, obstetric and anesthesiology care teams (Table 2) and distributed in both an electronic and paper copy to staff. The anesthetic plan was also forwarded to all obstetric anesthesiologists at our institution, with emphasis placed on invasive blood pressure monitoring with general anesthesia.

The patient presented in this case progressed rapidly after epidural catheter placement and received a brief epidural infusion of lidocaine amounting to a nontoxic level (e.g., 0.8 *μ*g/mL). Given the fact that neuraxial anesthesia has decreased the risks associated with anesthesia in the term parturient [15, 42], we aim to provide neuraxial techniques if there are no contraindications. However, parturients with Brugada syndrome should be offered the use of nitrous oxide (Nitronox™) or systemic opioids first for labor analgesia and be counseled on the treatment options. Although our patient was lower risk and elected for a CSE, it should be noted that an opioid-based regimen free of local anesthetic can be considered for regional anesthesia in higher risk patients.

In summary, Brugada syndrome poses an inherent risk of cardiovascular comorbidity and mortality, potentially more so in the peripartum period [43–45], and communication amongst all parties is essential for patient safety. Preoperative planning with the patient's cardiologist and obstetrician aided in development of an appropriate management strategy, with contingencies based on clinical progression. This case demonstrates that in parturients with rare diagnoses, the individual patient profile, various anesthetic techniques, and available literature should be considered prior to hospital admission. Last, regional anesthesia and local anesthetics can be administered safely to laboring patients with Brugada syndrome if precautions are utilized. When used briefly and in an admixture with epinephrine, lidocaine may be efficacious as an epidural infusion for labor analgesia.

## Figures and Tables

**Table 1:** Previous reports of central neuraxial anesthesia in parturients with Brugada syndrome, with the various medications and dosages used.

Author	Year published	Central neuraxial technique	Medications administered	Dose
*Kloesel* [24]	2011	Epidural	Sufentanil	5 *μ*g
			1% 2-Chlorprocaine	70 mg
			Fentanyl^*∗*^-0.125% bupivacaine	n/a
			Bupivacaine 0.125% (bolus)	12.5 mg

*Van de Knijff-van Dortmont* [22]	2016	Epidural	1 *μ*g/mL Sufentanil-0.1% ropivacaine	n/a

*Bramall* [19]	2016	Spinal	0.5% bupivacaine	13.5 mg
			Diamorphine	400 *μ*g

*Dawe* [25]	2018	Spinal	0.5% bupivacaine	12.5 mg
			Diamorphine	400 *μ*g

*Anaesthesia, (Abstracts)* [26]	2019	Spinal	Bupivacaine^*∗*^	1.5 mL
			Diamorphine	300 *μ*g

*Marques da Costa* [27]	2019	Combined spinal-epidural	0.75% ropivacaine	13.5 mg
			Sufentanil	2.5 *μ*g

*Ranji* [28]	2020	Spinal	Bupivacaine 0.75%	10.5 mg
			Fentanyl	15 *μ*g
			Morphine	150 *μ*g

*Sleipness* [21]	2020	Combined	2 *μ*g/mL Fentanyl-0.125%	n/a
		Spinal-epidural	ropivacaine	

^*∗*^Denotes an unknown concentration of the listed medication.

**Table 2:** Abbreviated list of medications and their safety profile for use in patients with Brugada syndrome.

Avoided or preferably avoided [37]	Drug-induced Brugada syndrome [38]	Potentially safe [39]
**Antiemetics**	**Antihistamines**	**Antiemetics**
Metoclopramide	Diphenhydramine	Dexamethasone [24]
Prochlorperazine	Terfenadine	Droperidol
		Ondansetron [24]
**Antiarrhythmics**	**Miscellaneous**	**Analgesics**
Flecainide	Alcohol	Ketorolac
Procainamid	Acetylcholine	Opioids
Propafenone	Cocaine	
	Propofol [31, 40, 41]	
**Local anesthetics**	**Selective serotonin**	**Anesthetics**
Bupivacaine	**Reuptake inhibitors**	Nitrous oxide
Lidocaine	Fluoxetine	Neuromuscular blockers
Procaine	Paroxetine	Reversal agents (*e.g., Neostigmine/Glycopyrrolate, Sugammadex*)
**Miscellaneous**		Thiopental Volatile agents (*e.g., Desflurane, Isoflurane, Sevoflurane*)
Cannabis		Volatile agents (*e.g., Desflurane, Isoflurane, Sevoflurane*)
Ketamine [32]		
Oxcarbazepine		
Tramadol		
**Psychotropics**	**Tricyclic antidepressants**	**Sedatives**
Amitriptyline	Doxepin	Midazolam
Clomipramine	Imipramine	
Lithium		
Loxapine		
Nortriptyline		
Trifluoperazine		
**Uterotonics**		
Methylergonovine		

## Additional Points

Preadmission and contingency planning for a third-trimester parturient with Brugada syndrome. Safe use of a lidocaine, epinephrine, and fentanyl epidural infusion for labor analgesia in Brugada syndrome. Reports of central neuraxial techniques for obstetric management of Brugada parturients. Medications and their safety profile for patients with Brugada syndrome.
